# The genetic etiology of body fluids on chronic obstructive airways disease

**DOI:** 10.1186/s12931-023-02661-6

**Published:** 2024-01-19

**Authors:** Zhangkai J. Cheng, Haojie Wu, Zhenglin Chang, Jiahao Cheng, Suilin Wang, Changlian Liu, Yanxi Zhang, Shiliang Xu, Qiongqiong Wan, JinWen Ron, Kemin Liu, Baoqing Sun

**Affiliations:** 1 Department of Clinical Laboratory, National Center for Respiratory Medicine, National Clinical Research Center for Respiratory Disease, State Key Laboratory of Respiratory Disease, Guangzhou Institute of Respiratory Health, The First Affiliated Hospital of Guangzhou Medical University, Guangzhou, 510120 Guangdong China; 2 Guangzhou Laboratory, Guangzhou International Bio Island, XingDaoHuanBei Road, Guangzhou, 510005 Guangdong Province China; 3 Guangzhou University of Chinese Medicine, Guangzhou, China

**Keywords:** COPD, Bodily fluids, Mendelian randomization, Genome-wide analysis

## Abstract

**Background:**

Numerous studies have documented significant alterations in the bodily fluids of Chronic Obstructive Pulmonary Disease (COPD) patients. However, existing literature lacks causal inference due to residual confounding and reverse causality.

**Methods:**

Summary-level data for COPD were obtained from two national biobanks: the UK Biobank, comprising 1,605 cases and 461,328 controls, and FinnGen, with 6,915 cases and 186,723 controls. We also validated our findings using clinical data from 2,690 COPD patients and 3,357 healthy controls from the First Affiliated Hospital of Guangzhou Medical University. A total of 44 bodily fluid biomarkers were selected as candidate risk factors. Mendelian randomization (MR) and meta-analyses were used to evaluate the causal effects of these bodily fluids on COPD and lung function (FEV1/FVC).

**Results:**

Mendelian randomization (MR) and meta-analyses, by integrating data from the UK Biobank and FinnGen cohort, found that 3 bodily fluids indicators (HDLC, EOS, and TP) were causally associated with the risk of COPD, two (EOS and TP) of which is consistent with our observational findings. Moreover, we noticed EOS and TP were causally associated with the risk of lung function (FEV1/FVC).

**Conclusions:**

The MR findings and clinical data highlight the independent and significant roles of EOS and TP in the development of COPD and lung function (FEV1/FVC), which might provide a deeper insight into COPD risk factors and supply potential preventative strategies.

**Supplementary Information:**

The online version contains supplementary material available at 10.1186/s12931-023-02661-6.

## Introduction

Chronic Obstructive Pulmonary Disease (COPD) is characterized by progressive, partially reversible airway obstruction accompanied by localized inflammation and systemic comorbidities [1, 2]. Currently, COPD is perceived as a composite disease comprised of chronic bronchitis, asthmatic bronchitis, and emphysema [3]. In 2019, the World Health Organization reported it caused approximately 3.23 million deaths and ranked seventh in global health impact based on Disability-Adjusted Life Years (DALYs). Notably, research progress has been limited over the last decade in identifying solutions and preventive measures for COPD [2]. The disease’s diverse pathophysiology and clinical features, which differ significantly among patients, contribute to its complexity, making treatment protocols challenging [4]. Recent studies have identified notable changes in the bodily fluids of COPD patients, indicating that understanding the disease’s origin and potential treatment strategies might be achieved through comprehensive analysis of these fluids [5, 6].

In the field of observational epidemiology, Mendelian randomization (MR) has emerged as an increasingly valuable tool for evaluating causal relationships [7]. The popularity of MR has surged over the past decade, primarily due to its innovative use of genetic variations as instrumental variables [8]. These genetic variations are employed to explore causal relationships between modifiable risk factors and health outcomes within observational data sets [9]. Compared to conventional observational studies, MR reduces confounding from environmental factors since allelic genes are randomly assigned and set during gamete formation and conception [10]. Additionally, MR addresses reverse causation bias as the allelic randomization precedes disease onset.

In the current study, we obtained the summary-level statistical data for 44 bodily fluid factors from large-scale GWAS and sought to infer potential causal associations of these candidate indicators on COPD. Subsequently, meta-analyses were applied to evaluate the combined causal effect of bodily fluid indicators on COPD. Lastly, we gathered clinical data on these candidate indicators from healthy individuals and COVID-19 patients to verify the MR findings.

## Methods

### Study design

**Fig. 1:**
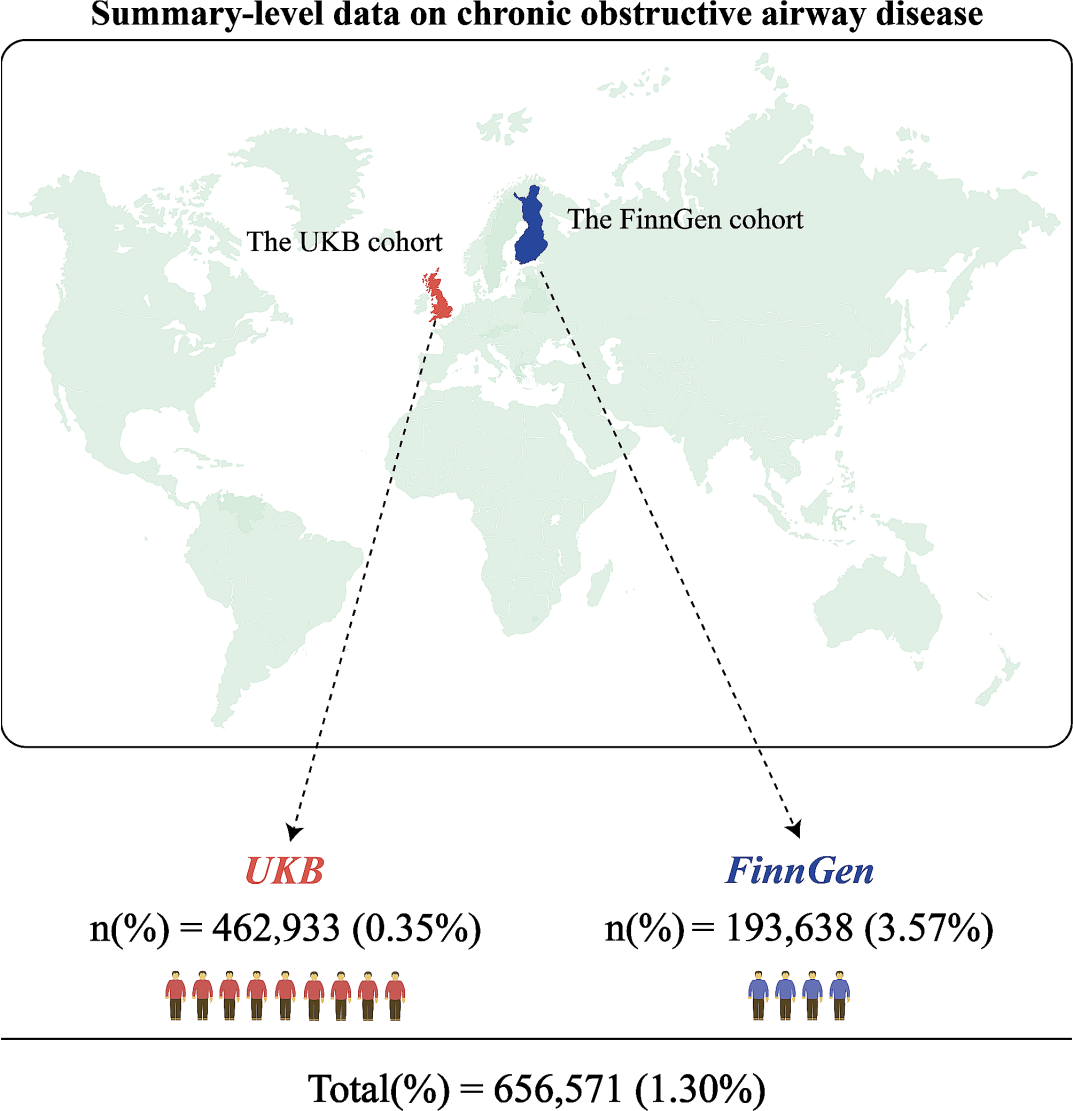
The geographical locations of three cohorts and summarization of the sample size (the proportion of cases) of MR studies

The two-sample Mendelian randomization (MR) analysis is an instrumental variable (IV) analysis extensively employed to assess the causal impact of exposures on outcomes using genetic variants as IVs of exposure [10–12]. Three fundamental assumptions underlie MR studies: Firstly, the genetic variants chosen as IVs must be strongly associated with the exposure (Assumption 1). Secondly, the selected variants should not be associated with any confounders (Assumption 2). Thirdly, the utilized variants should not be associated with the outcome except through the exposure (Assumption 3). Since this study involves a re-analysis of publicly accessible summary-level data from large Genome-Wide Association Studies (GWAS), no additional ethical approval was required. Meta-analyses were subsequently conducted to evaluate the combined causal effect. An observational study in China was used to verify the reliability and repeatability of the MR results. The workflow is displayed in Fig. 1.

### Collection of GWAS summary datasets

Summary-level data on chronic obstructive airways disease (COPD) were obtained from two nationwide biobanks: UK Biobank and FinnGen. Chronic obstructive airway disease was defined by J44 in the International Classification of Diseases, 10th Revision (ICD-10). A total of 44 bodily fluid markers were selected as candidate risk factors, and these were categorized into six major groups: hematological, kidney-related, liver-related, metabolic, urine, and other biochemical traits. The summary-level statistical European ancestry data for various factors such as white blood cell count (WBC), basophil cell count (BASO), etc., were downloaded from the Blood Cell Consortium [13, 14]. Data for factors like neutrophil count (NEU), red blood cell count (RBC), and others were sourced from the UK Biobank GWAS [15, 16]. Data for glomerular filtration rate (GFR) was sourced from a large mixed-population GWAS [17], and data for myoglobin levels (MB) were obtained from another large GWAS [18]. Data for lung function (FEV1/FVC) were obtained from a large GWAS [19].

A rigorous quality control procedure was initiated to identify eligible single-nucleotide polymorphisms (SNPs) that could serve as instrumental variables. Initially, SNPs with a genome-wide significance level (*P* < 5e-8) were collected. To estimate linkage disequilibrium (LD) between the SNPs, an LD clumping process was employed with criteria set at LD r2 < 0.001 and a clumping distance cutoff of 10,000 kb. To mitigate the risk of pleiotropy, we used Phenoscanner2 to check if any of the exposure-associated SNPs were correlated with known confounders of urolithiasis. Subsequently, SNPs that were not present in the outcome GWAS were removed, along with those that showed no significant association with the outcome (*P* > 5e-5). The final selection comprised more than three SNPs. Harmonization techniques were then applied to exclude palindromic and incompatible SNPs. To assess the potential for weak instrumental variable bias, we calculated the F statistic based on the formula F = (R^2 / (1 - R^2)) x ((n - k − 1) / k), where R^2 = 2 x MAF x (1 - MAF) x Beta^2, n represents the sample size, k is the number of instrumental variables, and MAF is the minor allele frequency [20].

### Observational studies in China

Clinical data pertaining to candidate risk factors were collected through a retrospective review of electronic health records at the First Affiliated Hospital of Guangzhou Medical University from 2021 to 2022. Our study included participants aged between 18 and 65 years. Those with missing information were excluded, resulting in a final analytical sample consisting of 2,690 COPD patients and 3,357 healthy controls. The demographic information of the participants is summarized in Table S1. Logistic regression was used to control for potential confounders and to generate odds ratios. Given the retrospective nature of our research, additional ethical approval was not required according to the Ethics Committee of the First Affiliated Hospital of Guangzhou Medical University.

### Statistical analyses

The TwoSampleMR package in R version 4.02 was used for conducting MR analyses. After harmonizing the effect alleles between the GWAS of exposures and COPD, the conventional fixed-effects inverse-variance weighted (IVW) method served as the primary statistical model for estimating causality. Estimates were then pooled using meta-analysis. In cases of low heterogeneity (I2 < 50%), a fixed-effects model was applied, whereas a random-effects model was used otherwise. The IVW method presupposes that the instruments influence the outcome solely through the exposure in question [21]. Additionally, MR-Egger and weighted-median methods were employed as supplements to IVW [22, 23]. Tests for directional pleiotropy using MR-Egger and Cochran’s Q statistics were also conducted.

## Results

**Fig. 2:**
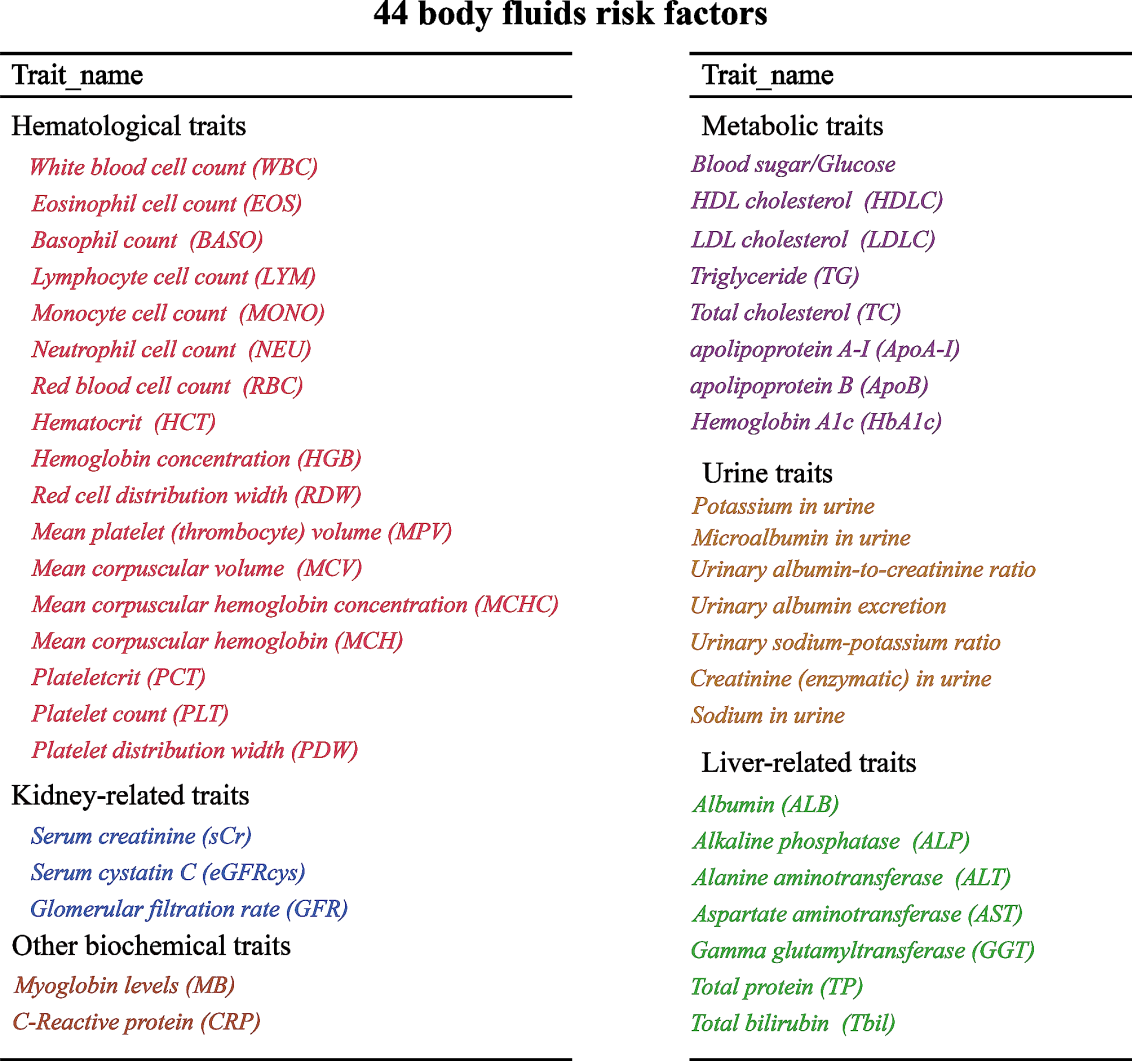
Summary of the peripheral markers selected from large-scale Genome-wide association studies

Summary-level data on COPD were sourced from two nationwide biobanks and included in our analyses (Table 1). The sample sizes and case proportions are depicted in Fig. 1. In the UK Biobank (UKB) cohort, 1,605 COPD cases and 461,328 controls were analyzed. In the FinnGen cohort, the numbers were 6,915 cases and 186,723 controls. We selected 44 bodily fluids markers as candidate risk factors (Fig. 2). The range of instrumental variables for these markers varied from 7 to 510. Almost all the markers had strong genetic instruments, with F statistics greater than 10 for 42 out of 44 selected factors (Supplementary Table 1). Detailed information on COPD independent SNPs (after the clumping process) for body fluids factors were listed in Supplementary Tables S2-7.

**Table 1:** Baseline characteristics of participants

	Healthy individuals	COPD patients	*P*
	*N*= 3357	*N*=2690	
Age[mean(SE)]	37.45 ± 11.12	58.45 ± 5.52	<0.001*
Gender(%)			<0.001*
Male	44.65%	92.19%	
Female	55.35%	7.81%	

*: Significantly different between healthy individuals and COPD patients (*p*<0.05).

Categorical variables are expressed as numbers (percentages).

Normally distributed continuous variables are presented as means ± SDs.

**Fig. 3:**
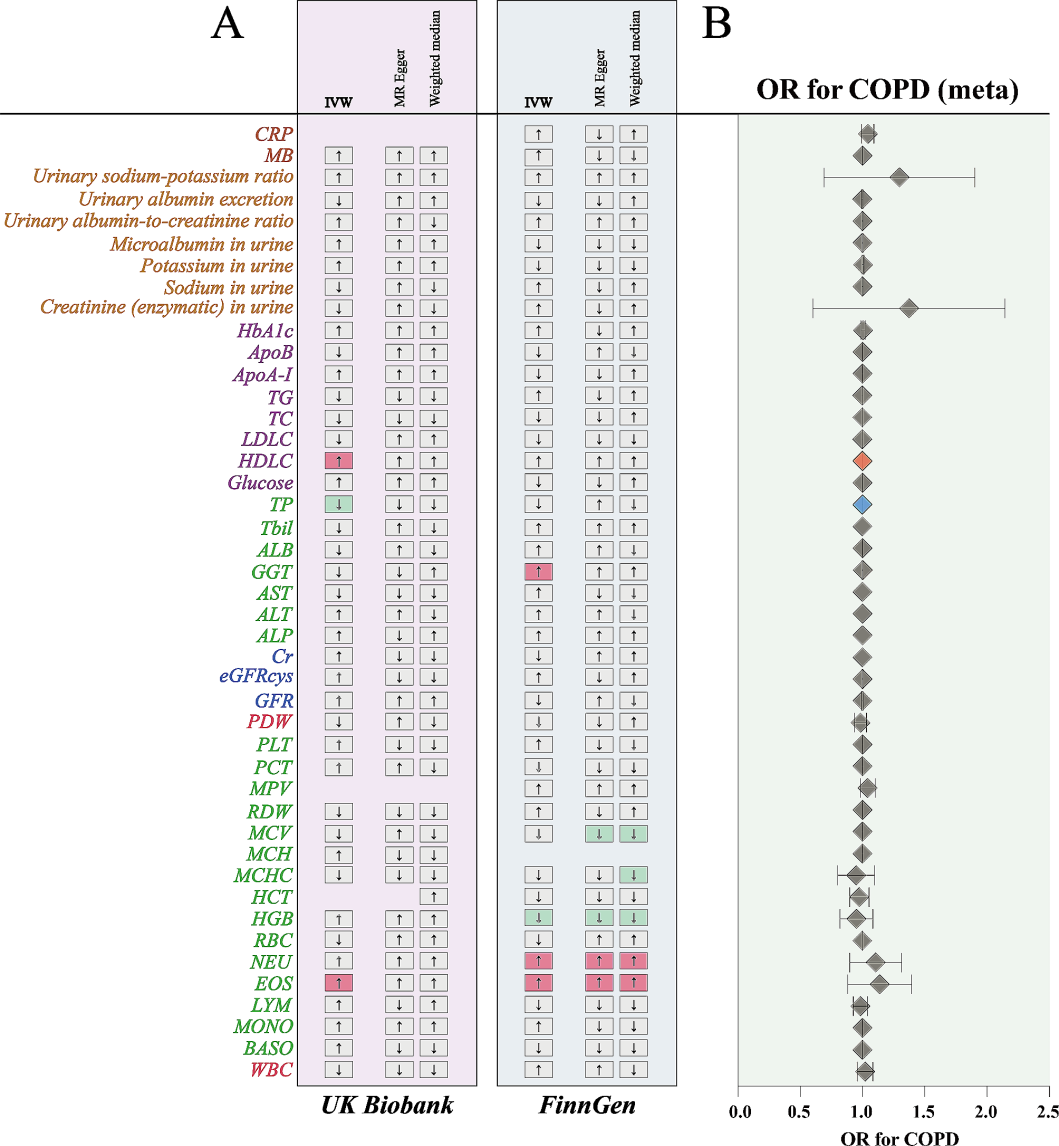
The causal association of candidate body fluid indicators on COPD. **(A).** Overview of the associations of 44 candidate markers on COPD. IWV indicates an Inverse variance-weighted method. **(B).** The meta-analysis of the association results from the two cohorts by the inverse-variance weighted method. The diamonds refer to the point estimates, and the horizontal bars represent the 95% confidence interval. The effect on the x-axis is the odds ratio of COPD per 1 standard deviation change in the exposure. The red diamonds indicated higher odds of COPD (*P* < 0.05), the blue diamonds indicated lower odds of COPD (*P* < 0.05), and the grey diamonds indicated the odds of COPD (*P* > 0.05)

In the UKB cohort, higher genetically-predicted levels of eosinophil cell count (EOS) and HDL cholesterol (HDLC) were significantly associated with increased COPD risk, whereas higher total protein (TP) levels were linked to decreased risk (Fig. 3A). The odds ratios (ORs) were 1.0010 (95% CI, 1.0004–1.0017) per a one-SD increase in EOS levels, 1.0010 (95% CI, 1.0001–1.0020) per a one-SD increase in HDLC levels, and 0.9997 (95% CI, 0.9994–0.9999) per a one-SD increase in TP levels (Supplementary Table S8).

In the FinnGen cohort, increased levels of EOS, neutrophil count (NEU), and gamma-glutamyltransferase (GGT) were significantly associated with higher COPD risk, while higher hemoglobin concentration (HGB) levels were associated with lower risk (Fig. 3A). The ORs were 1.2613 (95% CI, 1.1546–1.3778) per a one-SD increase in EOS levels, 1.2142 (95% CI, 1.0845–1.3595) per a one-SD increase in NEU levels, 1.0040 (95% CI, 1.0002–1.0079) per a one-SD increase in GGT levels, and 0.8658 (95% CI, 0.7549–0.9929) per a one-SD increase in HGB levels (Supplementary Table S9).

The meta-analysis indicated that HDLC (OR, 1.0010; 95% CI, 1.0001–1.0020) and TP (OR, 0.9997; 95% CI, 0.9994–0.9999) could influence COPD (Fig. 3B). Despite the meta-analysis failing to confirm the causality of EOS on COPD due to high inter-study heterogeneity (I2 = 96%), EOS emerged as a causal risk factor in both the UKB and FinnGen cohorts, warranting its consideration as a causal risk factor for COPD.

**Fig. 4:**
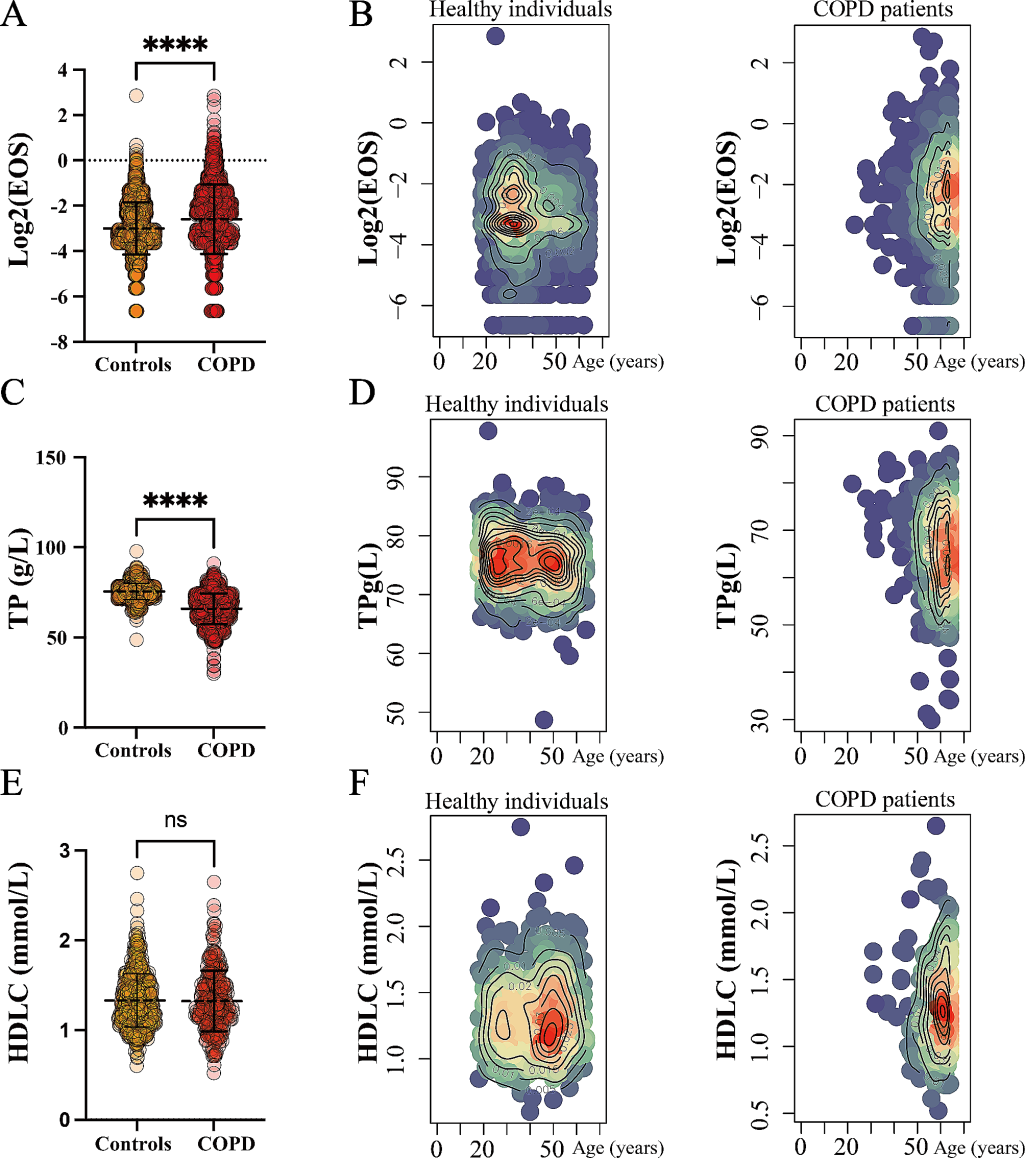
Difference of the clinical body fluid indicators between COPD patients and healthy individuals. **(A).** The relative expression of EOS in COPD patients and healthy individuals. **(B).** The density plot illustrates the distribution of EOS expression levels in healthy individuals compared to those with COPD. **(C).** The relative expression of TP in COPD patients and healthy individuals. **(D).** The density plot illustrates the distribution of TP expression levels in healthy individuals compared to those with COPD. **(E).** The relative expression of HDLC in COPD patients and healthy individuals. **(F).** The density plot illustrates the distribution of HDLC expression levels in healthy individuals compared to those with COPD.

To verify the reliability of our MR findings, we first collected the clinical data of 2690 COPD patients and 3357 healthy controls from the First Affiliated Hospital of Guangzhou Medical University (Table S1). In comparison with the healthy controls, the COPD patients were significantly older and predominantly male (Table 1). Subsequently, we evaluated three candidate indicators in COPD patients against those in healthy controls. The levels of EOS were found to be significantly higher in COPD patients as compared to the healthy controls (Fig. 4A-B). Additionally, levels of TP were significantly lower in COPD patients (Fig. 4C-D). In contrast, the levels of HDLC showed no significant difference between COPD patients and the controls (Fig. 4E-F).

Finally, to further assess the causal relationship between two candidate factors and the severity of COPD, we retrieved Genome-Wide Association Study (GWAS) data on lung function (FEV1/FVC ratio). Increased levels of EOS were significantly associated with higher COPD risk, while TP levels were associated with lower risk. The ORs were 0.9252 (95% CI, 0.9060–0.9449) per a one-SD increase in EOS levels and 1.0168 (95% CI, 1.0079–1.0258) per a one-SD increase in TP levels (Table S11).

## Discussion

Uncovering the causal relationship between bodily fluid indicators and Chronic Obstructive Pulmonary Disease (COPD) in extensive population datasets can offer invaluable insights for disease prevention. In present study, the FinnGen and UKB cohorts were applied to identify the causal risk indicators for COPD. Out of 44 potential indicators, we established the causal roles of three including HDLC, EOS, and TP through Mendelian Randomization (MR) and meta-analyses. Clinical data validation showed that two of these indicators (EOS and TP) align with our MR results. These discoveries could shed new light on the pathophysiology of COPD and pave the way for innovative diagnostic and therapeutic approaches.

Our research underscores EOS, a biomarker for Type 2 eosinophilic asthma, as a significant causative agent for COPD. Historically, the overlap between asthma and COPD, termed Asthma-COPD Overlap (ACO), has been recognized due to shared features [24]. However, the exact causality and sequence between the two remain elusive [25, 26]. A prospective study from as early as 2014 indicated that children with severe asthma, even without a history of smoking, are at a higher risk of developing COPD in their adult years [27]. Furthermore, prior research has observed an increase in eosinophilic granulocytes in induced sputum and bronchial biopsies during acute exacerbations [28], and elevated blood eosinophil levels have been linked to increased COPD mortality [29]. This suggests a possible causal relationship between EOS-driven inflammation and COPD. While further validation is needed, our findings spotlight EOS’s potential role in COPD treatment and research directions [30].

TP stands as a pivotal marker of nutritional health, and our initial findings underscore a pronounced decrease in TP among COPD patients. This decline is further emphasized by extensive research linking reduced body weight to heightened mortality and morbidity rates in these individuals [31–33]. Nutritional strategies have not only shown promise in enhancing energy and protein intake but also in shortening hospital stays and reducing readmission rates among other nutrition-centric outcomes for COPD patients [34, 35]. Furthermore, better nutrition correlates with significant improvements in respiratory muscle strength (MIP and MEP) and an uplifted health-related quality of life (HRQoL) for the malnourished with COPD [34]. Historically, the nuanced relationship between COPD, TP levels, weight loss, and cachexia was ambiguous. It was uncertain whether COPD’s progression triggered these conditions or if they played a role in COPD’s onset. Yet, our research supports the causal protective effect of TP on COPD.

The discovery that HDLC serves as a causative factor for COPD adds further credence to existing hypotheses within the academic community [36, 37]. Elevated HDLC levels in COPD patients have led to multiple interpretations: some believe these levels indicate a heightened COPD risk due to HDLC’s interaction with alpha-1 antitrypsin and pulmonary surfactants [38, 39]; others see it as a survival bias, given the co-existence of COPD with cardiovascular diseases (CVD) and the protective role of high HDLC against CVD [40, 41]. Another theory suggests HDLC directly contributes to COPD development by influencing prostaglandin synthesis and glycosaminoglycan secretion, which can impact lung function [42–44]. Our Mendelian Randomization analysis hints that elevated HDLC levels could predispose individuals to COPD. Still, further research, incorporating CVD variables, further clinical trials is crucial to validate these theories and guide targeted treatments.

Our in-depth analysis underscores that many of the identified risk markers have already been associated with COPD in scholarly literature. Interestingly, several of these risk factors seem to be interlinked. For instance, EOS and NEU, both inflammatory cells, exhibit a mutual relationship, even though they were distinctly analyzed in our study. Similarly, HDLC and TP, acting as pivotal metabolic indicators, demonstrate a multifaceted relationship with COPD beyond merely serving as risk markers. What sets our research apart is the systematic use of Mendelian Randomization (MR) to delve into the impacts of genetic etiology-induced fluid changes on COPD outcomes. This novel methodology led us to pinpoint three bodily fluid factors with significant implications for COPD. Furthermore, our results highlight pronounced variations in these risk factors across diverse populations, emphasizing the potential necessity for bespoke biomarkers.

In conclusion, our study sheds light on biomarkers relevant to COPD and lung function, yet we recognize it has limitations. The UK Biobank and FinnGen GWAS datasets utilized lack data on comorbidities, BMI, and specific medications like corticosteroids and statins, all of which can influence TP and HDLC levels. This omission might introduce potential biases in our results. Additionally, the demographic scope of our data may limit the generalizability of our findings across diverse populations. Lasty, indicators that exhibit differences in observational studies did not show causality in our analysis, suggesting the influence of confounding factors inherent in such study designs.

Despite these constraints, we hope our work will inform future research that can offer a more holistic view of biomarkers in COPD, taking into account a broader array of influencing factors and treatments.

### Electronic supplementary material

Below is the link to the electronic supplementary material.

Supplementary Material 1

Supplementary Material 2

Supplementary Material 3

Supplementary Material 4

Supplementary Material 5

## Data Availability

All data in this study are publicly available summary statistics. All accession numbers listed in Table 1 can be directly linked to their corresponding indicators by searching on the IEU OpenGWAS project (https://gwas.mrcieu.ac.uk).

## References

[1] Decramer M, Janssens W, Miravitlles M (2012). Chronic obstructive pulmonary disease. Lancet.

[2] The L (2022). COPD: from an end-stage disease to lifelong lung health. Lancet.

[3] Fishman AP (2005). One hundred years of chronic obstructive pulmonary disease. Am J Respir Crit Care Med.

[4] Singh D, Bafadhel M, Brightling CE, Sciurba FC, Curtis JL, Martinez FJ (2020). Blood Eosinophil counts in clinical trials for Chronic Obstructive Pulmonary Disease. Am J Respir Crit Care Med.

[5] Biljak VR, Pancirov D, Cepelak I, Popović-Grle S, Stjepanović G, Grubišić TŽ (2011). Platelet count, mean platelet volume and smoking status in stable chronic obstructive pulmonary disease. Platelets.

[6] Pavord ID, Chanez P, Criner GJ, Kerstjens HAM, Korn S, Lugogo N (2017). Mepolizumab for Eosinophilic Chronic Obstructive Pulmonary Disease. N Engl J Med.

[7] Haycock PC, Burgess S, Wade KH, Bowden J, Relton C, Davey Smith G (2016). Best (but oft-forgotten) practices: the design, analysis, and interpretation of mendelian randomization studies. Am J Clin Nutr.

[8] Burgess S, Small DS, Thompson SG (2017). A review of instrumental variable estimators for mendelian randomization. Stat Methods Med Res.

[9] Davies NM, Holmes MV, Davey Smith G (2018). Reading mendelian randomisation studies: a guide, glossary, and checklist for clinicians. BMJ.

[10] Chang Z, An L, Lei M, Song Z, Deng J, Tang R (2023). The genetic associations of COVID-19 on genitourinary symptoms. Front Immunol.

[11] Emdin CA, Khera AV, Kathiresan S, Mendelian Randomization (2017). JAMA.

[12] Smith GD, Ebrahim S (2003). Mendelian randomization’: can genetic epidemiology contribute to understanding environmental determinants of disease?. Int J Epidemiol.

[13] Chen MH, Raffield LM, Mousas A, Sakaue S, Huffman JE, Moscati A (2020). Trans-ethnic and ancestry-specific blood-cell Genetics in 746,667 individuals from 5 global populations. Cell.

[14] Vuckovic D, Bao EL, Akbari P, Lareau CA, Mousas A, Jiang T (2020). The polygenic and monogenic basis of Blood traits and diseases. Cell.

[15] Richardson TG, Sanderson E, Palmer TM, Ala-Korpela M, Ference BA, Davey Smith G (2020). Evaluating the relationship between circulating lipoprotein lipids and apolipoproteins with risk of coronary heart disease: a multivariable mendelian randomisation analysis. PLoS Med.

[16] Backman JD, Li AH, Marcketta A, Sun D, Mbatchou J, Kessler MD (2021). Exome sequencing and analysis of 454,787 UK Biobank participants. Nature.

[17] Pattaro C, Teumer A, Gorski M, Chu AY, Li M, Mijatovic V (2016). Genetic associations at 53 loci highlight cell types and biological pathways relevant for kidney function. Nat Commun.

[18] Folkersen L, Gustafsson S, Wang Q, Hansen DH, Hedman AK, Schork A (2020). Genomic and drug target evaluation of 90 cardiovascular proteins in 30,931 individuals. Nat Metab.

[19] Loh PR, Kichaev G, Gazal S, Schoech AP, Price AL (2018). Mixed-model association for biobank-scale datasets. Nat Genet.

[20] Papadimitriou N, Dimou N, Tsilidis KK, Banbury B, Martin RM, Lewis SJ (2020). Physical activity and risks of breast and colorectal cancer: a mendelian randomisation analysis. Nat Commun.

[21] Burgess S, Butterworth A, Thompson SG (2013). Mendelian randomization analysis with multiple genetic variants using summarized data. Genet Epidemiol.

[22] Bowden J, Davey Smith G, Burgess S (2015). Mendelian randomization with invalid instruments: effect estimation and bias detection through Egger regression. Int J Epidemiol.

[23] Bowden J, Davey Smith G, Haycock PC, Burgess S (2016). Consistent estimation in mendelian randomization with some Invalid instruments using a weighted median estimator. Genet Epidemiol.

[24] Morissette M, Godbout K, Côté A, Boulet L-P (2022). Asthma COPD overlap: insights into cellular and molecular mechanisms. Mol Aspects Med.

[25] Dasgupta S, Ghosh N, Bhattacharyya P, Roy Chowdhury S, Chaudhury K (2023). Metabolomics of asthma, COPD, and asthma-COPD overlap: an overview. Crit Rev Clin Lab Sci.

[26] Leung JM, Sin DD (2017). Asthma-COPD overlap syndrome: pathogenesis, clinical features, and therapeutic targets. BMJ.

[27] Tai A, Tran H, Roberts M, Clarke N, Wilson J, Robertson CF (2014). The association between childhood asthma and adult chronic obstructive pulmonary disease. Thorax.

[28] Saetta M, Di Stefano A, Maestrelli P, Turato G, Ruggieri MP, Roggeri A (1994). Airway Eosinophilia in chronic bronchitis during exacerbations. Am J Respir Crit Care Med.

[29] Hospers JJ, Schouten JP, Weiss ST, Rijcken B, Postma DS (1999). Asthma attacks with eosinophilia predict mortality from chronic obstructive pulmonary disease in a general population sample. Am J Respir Crit Care Med.

[30] Benson VS, Hartl S, Barnes N, Galwey N, Van Dyke MK, Kwon N. Blood eosinophil counts in the general population and airways disease: a comprehensive review and meta-analysis. Eur Respir J. 2022;59(1). 10.1183/13993003.04590-2020 PMC8756293 34172466

[31] Donahoe M, Rogers RM, Wilson DO, Pennock BE (1989). Oxygen consumption of the respiratory muscles in normal and in malnourished patients with chronic obstructive pulmonary disease. Am Rev Respir Dis.

[32] Lewis MI, Belman MJ (1988). Nutrition and the respiratory muscles. Clin Chest Med.

[33] Renzetti AD, McClement JH, Litt BD (1966). The veterans Administration cooperative study of pulmonary function. 3. Mortality in relation to respiratory function in chronic obstructive pulmonary disease. Am J Med.

[34] Ferreira IM, Brooks D, White J, Goldstein R (2012). Nutritional supplementation for stable chronic obstructive pulmonary disease. Cochrane Database Syst Rev.

[35] Collins PF, Stratton RJ, Elia M (2012). Nutritional support in chronic obstructive pulmonary disease: a systematic review and meta-analysis. Am J Clin Nutr.

[36] Tisi GM, Conrique A, Barrett-Connor E, Grundy SM (1981). Increased high density lipoprotein cholesterol in obstructive pulmonary disease (predominant emphysematous type). Metabolism.

[37] Reed RM, Iacono A, DeFilippis A, Eberlein M, Girgis RE, Jones S (2011). Advanced chronic obstructive pulmonary disease is associated with high levels of high-density lipoprotein cholesterol. J Heart Lung Transplant.

[38] Florentin M, Liberopoulos EN, Wierzbicki AS, Mikhailidis DP (2008). Multiple actions of high-density lipoprotein. Curr Opin Cardiol.

[39] Ortiz-Muñoz G, Houard X, Martín-Ventura JL, Ishida BY, Loyau S, Rossignol P (2009). HDL antielastase activity prevents smooth muscle cell anoikis, a potential new antiatherogenic property. Faseb j.

[40] Huiart L, Ernst P, Suissa S (2005). Cardiovascular morbidity and mortality in COPD. Chest.

[41] Sin DD, Man SFP (2008). Impact of cancers and cardiovascular diseases in chronic obstructive pulmonary disease. Curr Opin Pulm Med.

[42] Fleisher LN, Tall AR, Witte LD, Miller RW, Cannon PJ (1982). Stimulation of arterial endothelial cell prostacyclin synthesis by high density lipoproteins. J Biol Chem.

[43] Pomerantz KB, Tall AR, Feinmark SJ, Cannon PJ (1984). Stimulation of vascular smooth muscle cell prostacyclin and prostaglandin E2 synthesis by plasma high and low density lipoproteins. Circ Res.

[44] Wosu L, Parisella R, Kalant N (1983). Effect of low density lipoprotein on glycosaminoglycan secretion by cultured human smooth muscle cells and fibroblasts. Influence of serum concentration and cell proliferation rate. Atherosclerosis.

